# SARS-CoV-2 epidemiology and control, different scenarios for Turkey

**DOI:** 10.3906/sag-2003-260

**Published:** 2020-04-21

**Authors:** Eskild PETERSEN, Deniz GÖKENGİN

**Affiliations:** 1 ESCMID Emerging Infections Task Force, European Society for Clinical Microbiology and Infectious Diseases, Basel Switzerland; 2 Directorate General for Disease Surveillance and Control, Ministry of Health, Muscat Oman; 3 Institute for Clinical Medicine, University of Aarhus, Aarhus Denmark; 4 Department of Infectious Diseases and Clinical Microbiology, Faculty of Medicine, Ege University, İzmir Turkey

**Keywords:** COVID-19, Coronavirus, epidemiology, control, mortality

## Abstract

**Background/aim:**

Coronavirus Infectious Disease 2019 (COVID-19) is now a pandemic spreading in most countries including Turkey.

**Materials and methods:**

The current knowledge of COVID-19 and the virus causing it, SARS-CoV-2, was reviewed. The epidemiology and control in different countries was compared and the differences discussed.

**Results:**

The population attack rates and case fatality rates vary from country to country with Lombardy in northern Italy reporting an attack rate in the general population of 0.37% compared to 0.004% in Hong Kong. The differences are caused by different testing strategies and reporting systems.

**Conclusion:**

Turkey is early in the outbreak. Different control strategies are available with South Korea, Hong Kong and Singapore being models to follow.

## 1. Introduction

The World Health Organization (WHO) declared the COVID-19 outbreak caused by the SARS-CoV-2 virus a pandemic on the 11th March [1].

Currently China, South Korea, Singapore and Hong Kong seems to have the pandemic under partial control with sporadic cases, but the number of cases is currently increasing rapidly in most European Union (EU) countries and the United States (US) [2].

Starting in Wuhan, Hubei Province, China, the rate of global dissemination has accelerated, and community spread is ongoing in many countries and as per the 27th March there were 509,164 confirmed cases and 23,335 deaths worldwide [2].

Containment is no longer a realistic goal in Europe and the US, and radical and urgent efforts are needed to mitigate the spread of infection, to avoid overwhelming health care systems. Without urgent action, the impact of this pandemic stands to become unprecedented in recent history [3]. Here, we summarize key epidemiological characteristics of the emerging SARS-CoV-2 and try to forecast the challenges faced by Turkey.

### 1.1. The 2003 SARS-CoV versus SARS-CoV-2

At the height of the 2003 SARS epidemic, 140 new infected patients were reported weekly [4]. SARS-CoV had a mortality rate of 9.7% and the majority of infections were nosocomial. The SARS epidemic ceased in 2003 within the year with a total of 8,098 reported cases with 774 deaths globally [5].

A clear contrast to SARS-CoV is the rate of expansion of the SARS-CoV-2 epidemic [6].The expert mission of the WHO to China concluded that “transmission of SARS-CoV-2 is mostly driven by clusters in close contacts, particularly family clusters, and less so community transmission” [7]. In addition, presymptomatic transmission takes place up to the start of symptoms [8] also in children [9,10]. 

The cases started to climb in Italy on 22nd February with 9 cases reported (3 cases on the 21st) and it can be argued that earlier mitigating measures could have prevented the steep rise in cases up to 86,498 with 9,134 deaths by the 28th March with no signs of the peak being reached yet [11]. In the United States, cases started to rise in late February and have rapidly escalated upwards with 68,334 confirmed cases reported by 26th March [2].

### 1.2. Why does SARS-CoV-2 spread so much more widely than SARS? 

SARS-CoV-2 has a high viral load at the onset of symptoms that declines up to 5–6 days later [12,13]. In comparison, for SARS-CoV excreted viral loads peaked at 6–11 days after onset [14,15], which makes isolation and quarantine of symptomatic individuals infected with SARS-CoV-2 much more challenging and less effective. Severe cases of COVID-19 have higher viral loads and excrete virus longer than mild cases [16]. Furthermore the SARS-CoV-2 spike protein has a high affinity for the receptor on human cells, the Acetylcholin-esterase-2, ACE2 [17]. 

#### 1.2.1. Incubation period

An early study estimated the mean incubation period to be 5.8 (95% confidence interval-CI 4.6 – 7.9,) days, ranging from 1.3 to 11.3 days [18].Another study estimated the median incubation period to be 5.1 days (95% CI, 4.5 to 5.8 days), and found that 97.5% of those who developed symptoms did so within 11.5 days (95% CI, 8.2 to 15.6 days) of infection [19].The average viral load in a study of seven subjects in a family cluster was 6.76 × 105 copies per swab during the first 5 days and live virus isolates were obtained from swabs during the first week of illness [20].

#### 1.2.2. Population immunity to the emerging virus

Pandemic of influenza (1918, 1957, 1968, 2009) spread in populations with little immunity [21,22]. A key difference between SARS-CoV-2 and pandemic influenza is the age distribution of cases. Children rarely have severe clinical illness but the infection attack rate in a household study was similar for children and adults [23]. The Korean government made a decision on school closure on 23rd February 2020, when there were signs of community spread [24]. The Korean response also included extensive testing and the outbreak in and up to the 28th March 2020, 387,925 tests were performed, 9,478 cases were identified of which 144 died. The epidemic curve in Korea is shown in Figure [24]. It is probably too early to determine the role of children in the spread of SARS-CoV-2 but the data so far support that the infection may be spread by children with few or no symptoms [24]. 

**Figure 1:**
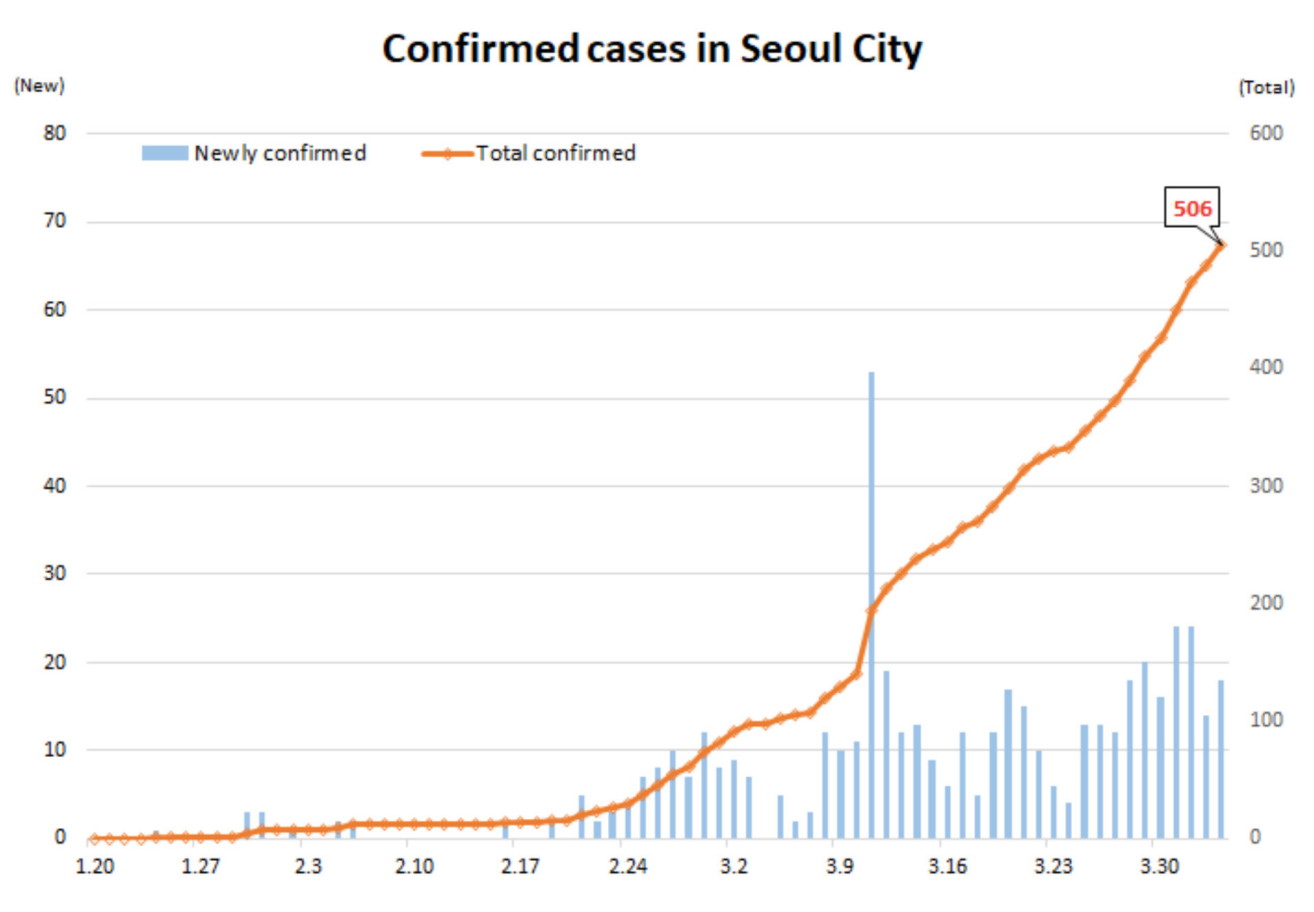
The Pandemic in Seoul, South Korea (Korea CDC) [24].

#### 1.2.3. Transmissibility – R0

Various models applied to early SARS-CoV-2 epidemic data found that an infected person spread the disease to an average of 2.6 people, the basic reproductive rate *R*0. Thus, after 10 generations of transmission, with each taking about 5–6 days, a single case would expand to more than 3,500 new cases in the course of just two months in the absence of mitigation strategies.

*The R*0 for SARS was estimated to be around 3 in the absence of control measures [5].

The *R*0 values of SARS-CoV-2 has been estimated to be 2.9 (95% CI: 2.3–3.7) [16]. A later study estimated the *R*0 to be 3.6 [25]. The *R*0 values have important implications for disease control [26]. The transmission has to be reduced below 1 *R*0 to ensure extinction of the disease. At *R*0 = 2.2 the fraction not transmitting the disease to reduce *R*0 to below 1 is 55% [26]. However, consensus is that rigorous mitigation measures are needed early on in order to slow down SARS-CoV-2 transmission [27].

For the 2009 A(H1N1)pdm influenza pandemic, initial estimates of *R*0 was1.7 [28]. For the 1918 pandemic, *R*o was estimated in the first wave at around 2 [21]. Even the *R*0 for the 2003 SARS in the beginning was estimated to be between 2 and 4 [29]. In contrast, MERS-CoV has a very high case fatality (34%) and low transmissibility. Since 2012, MERS-CoVhas caused 2,494 reported cases and 858 deaths in 27 countries. MERS-CoV has also caused some nosocomial outbreaks, mainly in hospitals in Saudi Arabia, Jordan, and South Korea. However, estimates of MERS-CoV R0 are less than 1, and thus far it has been contained [30].

## 2. Excess mortality from influenza and Covid-19

Clinical case fatality rates based on case definitions used in the Wuhan outbreak (fever and respiratory syndrome including pneumonia) are around 5% in the Hubei Province but far lower in the rest of China and South Korea, around 1.0% [31]. The latest data from Lombardy from the 27th March show that 30% of known cases are in hospital and 4.3% are in intensive care units (ICU) equivalent to 16 per 100,000 population [11]. Lombardy has a population of 10 million and the population attack rate as of 21st March is 0.37% and the population mortality rate is 45 per 100,000 [11].

A study of the 1957 influenza A(H2N2) pandemic found an excess respiratory mortality rate of 19 per 100,000 population (95% CI, 12-26 cases per 100,000 population) on average for the entire three year pandemic period of 1957–1959 [32]. The global mortality estimate was moderate relative to that of the 1918 pandemic but was approximately 10-fold greater than that of the 2009 pandemic [32].

A study from Denmark of the 2009 A(H1N1) influenza reported that the proportion of beds used for pandemic influenza cases did not exceed 4.5% of the total national ICU bed capacity [33]. Hospitals with cases used a median of 11% of ICU beds for pandemic influenza patients (range: 3%–40%). The population attack rate peaked at 5% in the week of 22 November 2009 [34].

### 2.1. Cumulative incidence and mortality from SARS-CoV-2

The numbers reported from the epicenter in China, Hubei province (59.170 million population) were 67,466 confirmed cases and 2,902 deaths, equal to a cumulated population attack rate of 0.11% and a cumulated fatality rate of 4.8 per 100,000 population [30]. This is far lower that the currently recorded fatality rate in Lombardy, Italy, of 44 per 100,000. A different age structure of the population cannot explain the ten-fold higher mortality in Lombardy (Table 1) and underreporting in China is possible.

**Table 1:** Estimated number of deaths in İstanbul if attack rates in different countries and areas are applied. Turkey has a population over 70 years of 5.86.9% (2019) [39].Estimated number of deaths in İstanbul if attack rates in different countries and areas are applied. Turkey has a population over 70 years of 5.86.9% (2019) [39].

	Attack rate%	Population over 65-70 years (%)	Deaths per 100,000
Lombardy, Italy [11]	0.34	22.5	25.2
Hubei province, China[31]	0.11	10.1	5.2
South Korea [24]	0.017	13.5 *	0.2
Singapore [37]	0.007	10.1 **	0.03
Hong Kong [2]	0.004	14.2 ***	4.3

* South Korea Population (2018). Worldometers [online]. Website www.worldometers.info [accessed 27th March 2020]. ** Singapore Department of Statistics (2020). Population and population structure [online]. Website https://www.singstat.gov.sg/find-data/search-by-theme/population/population-andpopulation- structure/latest-data [accessed 27 March 2020]. *** United Nations Statistics Division (2020). Demographic and Social Statistics [online] Website https://unstats.un.org/unsd/demographic-social/ [accessed 27 March 2020].

The critical question in the interpretation of these findings is what the extent of the epidemic has been, when including mild and asymptomatic cases [6].

The first case in Turkey was reported on March 10th, much later than in many European countries. The case had a history of travel from Europe. On March 12th, 4 new cases were reported with a steep rise to 2,433 out of 33,004 tested (7%) within 14 days as of March 25th with a death rate of 2.4% (n = 59). The daily number of tests was reported to be 5035 on day 14 and by the 27th March 3629 were recorded by the WHO with 75 deaths. On day 24 of the epidemic, the total number of tests was 125,556, positive cases was 18,135, with 356 deaths. A total of 1,101 cases were hospitalized in the ICU out of which 783 were intubated. İstanbul has the highest number of cases (n = 8852) compared to others followed by İzmir (n = 853), Ankara (n = 712) and Konya (n = 584) [35]. Detailed information is not available on gender, age, clinical stage and prognosis of the identified cases. The start of the epidemic shows great similarity with the rapidly rising trend observed at the early stages in Italy and Spain. Although the health authorities and the government in Turkey were quick to take action by cancelling schools and social, cultural, sports activities and scientific meetings, a nationwide lockdown was not put into practice. A rough estimation of the number of ill people and the number of deaths for Istanbul (20 million inhabitants) according to different scenarios from different countries and provinces (Table 1) shows that the consequences of an epidemic similar to that in Italy would be devastating.

In Table 2, we have tried to estimate the impact of the pandemic in Istanbul using numbers from Hubei (China), Lombardy (Italy), South Korea, Singapore and Hong Kong. 

**Table 2:** Estimating number of ill people and number of deaths in İstanbul (20 million inhabitants) according to different scenarios from different countries and provinces.

Scenario	Number of illpeople in İstanbul	Number ofdeaths
Lombardy (Italy)	54,000	5,060
Hubei (China)	22,000	1,043
South Korea	3,400	40
Singapore	1,400	6

### 2.2. What have other countries done?

**Korea**

The development of the SARS-CoV-2 infection illustrates how rapid the numbers increase in Figure. The numbers went from 2 daily cases to 909 from the 18th February to the 29th February. Extensive testing was applied to cases and contacts and a total of 387,925 tests or up to 10,000 per day were run to identify cases and contacts and quarantine them [24].

**Singapore**

Singapore was one of the worst affected areas in the 2003 SARS outbreak, and since then Singapore has steadily built up its outbreak preparedness, including developing a national pandemic preparedness plan based on risk assessment and calibration of response measures that are proportionate to the risk [36]. 

This includes holding regular exercises, and building the National Centre for Infectious Diseases (NCID), a 330-bed purpose built infectious diseases management facility with integrated clinical, laboratory and epidemiologic functions.

Some data from Singapore are shown in Table 1. Up to the 27th March, Singapore had reported a total of 683 cases with 2 deaths. The Ministry of Health (MOH) had developed a local case definition already by the 2nd of January 2020 and SARS-CoV-2 real-time polymerase chain reaction (RT-PCR) laboratory testing capacity was scaled up rapidly to all public hospitals in Singapore to handle 2,200 tests a day. All contacts were assessed by telephone for fever or respiratory symptoms by public health officials during the quarantine or monitoring period, thrice daily for close contacts and once daily for contacts at lower risk.

In late January 2020 the following groups were tested for COVID-19: 

1) all hospitalized patients with pneumonia (later expanded to include patients with pneumonia evaluated in primary care settings);

2) ICU patients with possible infectious causes as determined by the physician; 

3) patients with influenza-like illness at sentinel government and private primary care clinics included in the routine influenza surveillance network; and 

4) deaths from possible infectious causes [37].

## 3. Do interventions matter and what can we do?

**T**he pandemic due to the SARS-CoV-2 virus has caused high morbidity and mortality in the elderly, much higher than influenza. However, in contrast to influenza, children seem to be less severely affected.

The tools we have available with a new infection causing a pandemic including no specific treatment such as antivirals, no vaccine and a nonimmune population are no different than the tools available during pandemics of the past century and going back as far as to plague in the 14th century: quarantine and mobility restrictions.

A study of the mortality in 17 US cities during the 1918 pandemic found that cities in which multiple interventions were implemented at an early phase of the epidemic had a 50% lower peak mortality than those that did not and a 20% lower mortality rate in the course of a delayed, flatter epidemic curve [38].

This clearly demonstrates that mitigating policies are of paramount importance and make a difference, not only by lowering mortality, but also ensuring that the burden on the health care system remains manageable. 

Thus, social distancing and home quarantine should be practiced in countries with local transmission. This is urgent, as the window of opportunity is small. The examples of China, Singapore and some initial success in Korea show that it is possible to influence the spread of SARS-CoV-2, but that the societal and economical costs will be enormous and long-lasting. 

**Implementations already done in Turkey are:**

· School closure as children with few symptoms may contribute to community spread (1 day after the first case);

· Prohibiting public gathering and cancelling all public events like football matches, theater, cinema, religious gatherings (1 to 5 days after the first case);

· Ensuring that the health care system is prepared by revising protection of health care workers (procedures and use of personal protective equipment-PPE);

· Revising the available ICU capacity and being prepared to cancel elective surgery needing ICU backup;

· Isolating cases that do not need hospitalization and quarantine all contacts. 

**Still recommendable options available for Turkey are: **

· Continue to ensure a large testing capability with rapid availability of results. At the height of the outbreak in South Korea 10,000 test were done daily (reached after several weeks over 20,000 daily in Turkey);

· Continue to ensure procurement of an adequate and timely supply of PPE in each hospital;

· Provide a healthy work environment and reasonable working hours for healthcare providers;

· Consider taking advice and learning from countries like Korea, Singapore and China which has by now managed to control the pandemic in their respective countries. 

## Disclaimers

No funding has been received for this study.
